# Progesterone receptor isoforms A and B: new insights into the mechanism of progesterone resistance for the treatment of endometrial carcinoma

**DOI:** 10.3332/ecancer.2013.381

**Published:** 2013-12-18

**Authors:** Ruijin Shao

**Affiliations:** Department of Physiology/Endocrinology, Institute of Neuroscience and Physiology, The Sahlgrenska Academy, University of Gothenburg, Gothenburg 40530, Sweden

**Keywords:** progesterone receptor isoforms, progesterone resistance, cell—cell interaction, endometrial carcinoma, animal model

## Abstract

Progesterone therapy is an effective treatment for atypical endometrial hyperplasia and early endometrial carcinoma (EC). However, progesterone resistance is the main obstacle to the success of conservative treatment in women with type I EC and remains a major clinical challenge. Studies indicate that progesterone and progesterone receptors (PRs) play a significant role in both normal and neoplastic endometria. Most EC arises in the epithelial cells of the endometrial glands, and a large body of *in vitro* evidence suggests that the absence or reduced expression of PR isoform B might result in the failure of progesterone treatment and lead to aberrant PRB-mediated signalling in EC cells. A recently developed *in vivo* knockout mouse model suggests that enhanced DNA methylation decreases the level of stromal PR isoform A and that this is also a main contributor to progesterone resistance in EC cells. The endometrial stroma within the EC might create a microenvironment that determines how epithelial-derived cancer cells respond to progesterone. This novel study opened a new avenue for research seeking to clarify the mechanisms that regulate the specific PR isoforms that are associated with the stromal cell responses to progesterone and has led to new understanding of both endometrial cell-specific and mechanical contributions of the stroma to EC development.

## Introduction

Endometrial cancer (EC) remains the most common gynaecological malignancy in women across the globe [1, 2]. It has been reported that 7950 of the 43,470 women in the United States with diagnosed EC died in 2010 [3], and there are 1900 deaths per year from EC in the United Kingdom (http://www.cancerresearchuk.org). EC can be diagnosed early, but it remains difficult to manage with non-surgical interventions. Surgical procedures are still the first line and most effective treatments for the early stage of this disease [4]. There are numerous studies that describe human culture methods using several EC cell lines for the study of EC biology. However, a fundamental question remains as to how closely EC cell lines are able to recapitulate the biology of human EC *in vivo*. Co-culture experiments using EC cell line and human endometrial stromal cells designed for further understanding of the development of EC and interaction between normal tissue/stroma and cancer cells are lacking. Although our understanding of the pathophysiology of EC has improved, the results of potential preventive and therapeutic options, especially non-surgical treatments, have been disappointing.

Epidemiological studies have implicated steroid hormonal imbalance in the development of EC [5]. Continuous exposure of the endometrium to oestrogens can lead to endometrial overgrowth and hyperplasia [6], and progesterone acts as a protective factor against oestrogen-driven growth and proliferation in the endometrium [1]. Under pathological conditions, the survival and proliferation of EC cells can be suppressed by the actions of progesterone and its analogues, such as medroxyprogesterone acetate [7] and megestrol acetate [1]. Because of this dependence, treatment with progesterone and its analogues is the major non-surgical treatment for EC [8]. Such therapy is effective for atypical endometrial hyperplasia and early EC in many women who desire to maintain their fertility [9]. However, more than 30% of women with oestrogen-dependent, well-differentiated type I EC fail to respond to progesterone treatment due to progesterone resistance [10], and the underlying mechanisms behind this are still poorly understood.

## Discussion

It is well accepted that progesterone responsiveness in the endometrium [11] is mediated by the coordinated actions of progesterone receptor (PR) isoforms A and B. PR is a member of the steroid receptor super family that regulates transcription of numerous target genes [12]. Both PRA and PRB are transcribed from two different promoters in a single gene, and PRA differs from PRB by the absence of the 164 amino acids at the amino terminus of the protein [13]. The PRA and PRB isoforms have different activities and functions. For example, *in vitro* experiments show that PRA functions as a transcriptional inhibitor of PRB when PRA and PRB are both present in the same cells [13]. In addition, selective ablation of PRA, but not PRB, results in mouse uteri that fail to display progesterone-mediated inhibition of oestrogen-induced epithelial cell proliferation [14]. These results suggest that the distinct expression of PRA and PRB in the endometrium is likely to have different functional consequences [15]. In normal human endometria, PRA and PRB are both expressed in the epithelial and stromal cells [16], and both isoforms appear to fluctuate in the cycling endometrium in an isoform-specific and cell-specific manner [17, 18].

There is conflicting and contradictory clinical evidence regarding the use of PR isoform expression or the ratio of the two PR isoforms as a predictor of EC risk and prognosis [19–24]. However, the available data make it quite clear that the loss or downregulation of either one or both of the two PR isoforms in EC tissues is associated with higher clinical grade [24–26]. Regulation of PR expression is involved in several different processes including transcription, translation, and post-translational modification [1, 8]. *In vivo* studies with human EC tissues and *in vitro* studies with several EC cell lines have shown that epigenetic mechanisms such as DNA methylation and histone modification play crucial roles in regulating the total PRA and PRB expression [27–30].

**Figure 1.**
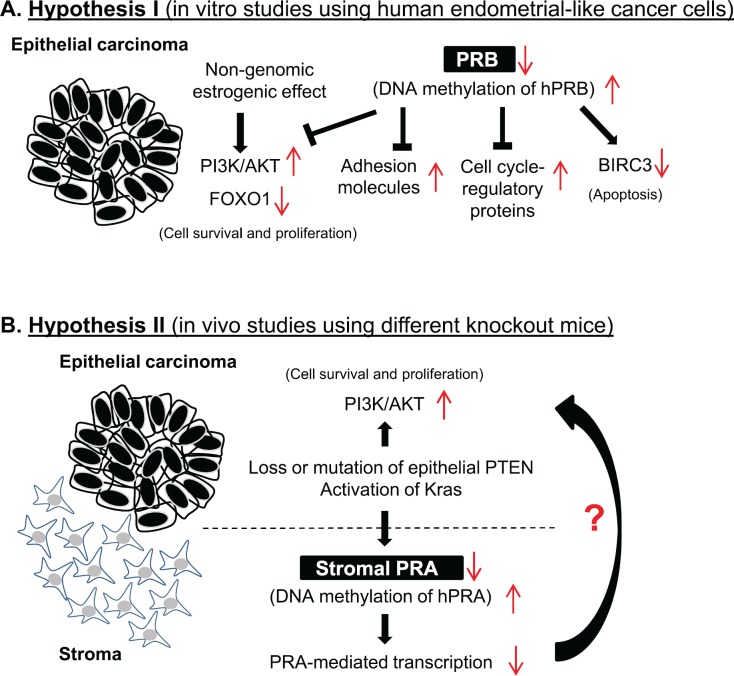
Two hypotheses have been developed to describe how endometrial cancer cells survive and proliferate by switching from progesterone sensitivity to progesterone resistance. Both of these hypotheses depend on the fact that transcription factors activated by progesterone receptor isoforms A and B play a central role in controlling cell proliferation, differentiation, and apoptosis in the endometrium under pathological conditions. *In vitro* studies using human endometrial cancer cells indicate that decreased PRB expression in endometrial cancer cells is likely responsible for progesterone treatment failure (A). Janzen *et al* used different knockout mouse models to show for the first time that the endometrial stromal component is also responsible for progesterone sensitivity and resistance, and that PRA is a critical factor mediating endometrial cellular response to progesterone treatment in endometrial cancer tissues *in vivo* (B).

There is no *in vivo* evidence for individual roles of the PR isoforms in the initiation and development of EC, but it has become progressively more evident from *in vitro* studies with human EC cell lines that activation of the phosphatidylinositol 3-kinase (PI3K)/protein kinase B (AKT) pathway [31, 32], alteration of adhesion molecules [33], and activation of the cell cycle-regulatory proteins [34] required for cell proliferation and apoptosis are most likely a result of PRB activity. These studies are further supported by the fact that significant alterations of forkhead box O1 (FOXO1), an AKT downstream effector, and baculoviral IAP repeat containing 3 (BIRC3), a PRB-regulated protein, are induced by progesterone treatment in these cells [32, 34]. Furthermore, the altered response of EC cells to progesterone therapy is probably due to changes in the level of PRB between pre-treatment and post-treatment with medroxyprogesterone acetate [35]. These observations thus have led to the proposal that decreased PRB expression in EC cells could be responsible for progesterone treatment failure (Figure 1A). It should be noted, however, that the use of *in vitro* culture systems with the different EC cell lines to study the specific PR isoform-mediated effects on progesterone response might fail due to the absence of the *in vivo* conditions under which EC develops.

EC arises most commonly in the epithelial cells of endometrial glands [1, 2], but the human endometrium also includes other cell types such as stromal fibroblastic cells in the stroma [15]. A number of studies suggest that the stroma component is not only supportive of tumour growth but can also be a causative factor for the initiation and development of many human cancers [36]. Insulin-like growth factor binding protein-1 (IGFBP-1) in stromal cells is one such causative candidate. Several studies have shown that oestrogen-mediated insulin-like growth factor-1 (IGF-1) synthesis in endometrial epithelial cells [37] is inhibited by progesterone-stimulated IGFBP-1 expression in endometrial stromal cells [37, 38]. IGF-1 receptors are present in the endometrial epithelial cells [38], and activation of IGF-1 signalling is an important event in the development of endometrial hyperplasia and EC [39]. Previous tissue recombination experiments have demonstrated that stromal PR is required for the inhibition of oestrogen-induced epithelial cell proliferation in the mouse uterus [40]. These data suggest that aberrant paracrine regulators released from stromal cells might promote epithelial proliferation, endometrial hyperplasia, and the progression of EC.

Several molecules have been shown to have a temporal and/or cell-specific expression pattern in the endometrium under physiological and pathological conditions [2, 9]. Dominant loss-of-function alterations in phosphatase and tensin (PTEN) homolog, a well-defined tumour suppressing molecule, is associated with the development and onset of human EC [31], and experimental manipulation of endometrial PTEN levels in mice verifies this observation [41]. It has been shown that the phosphatase activity of PTEN is required for inhibition of the PI3K/AKT signalling pathway [42], and this suggests that decreased PTEN-dependent up-regulation of the PI3K/AKT pathway plays a role in tumour progression [43].

Although studies using PTEN knockout mice demonstrate uterine tumour growth [41], the interpretation of the loss of PTEN has been complicated by the inability to differentiate between epithelial and stromal cell defects. In the 1 August 2013 issue of *Cancer Research*, Janzen *et al* [44] revealed that mice in which PTEN was specifically deleted in epithelial cells exhibited uterine tumour growth similar to what is seen in women with EC [31], and that the anti-tumour effects of progesterone depend on uterine cell-specific PR expression and regulation.

The significance of the article by Janzen *et al* [44] is threefold. First, it provides the strongest evidence that time-dependent systemic treatment with progesterone in epithelial-PTEN knockout mice is capable of significant suppression of tumour cell proliferation and the induction of tumour resolution. This inhibitory effect is likely mediated by stromal PR expression because ablation of stromal PR, but not epithelial PR, makes the uterine tumours in epithelial-PTEN knockout mice resistant to progesterone therapy. Although it is still not clear how stromal cells create their microenvironment and promote PR signalling to reach the adjacent tumour cells, one can assume that stromal PR-mediated regulation of molecules such as IGFBP-1 [37, 38] contributes to the inhibitory effects of progesterone in uterine tumours via a paracrine mechanism.

Second, Janzen and colleagues found that the efficiency of progesterone treatment in mouse uterine tumours requires the presence of oestrogen. There is evidence that oestrogen can upregulate uterine PR expression in rats *in vivo* [45] and in most progesterone target cells *in vitro* [11]. These results suggest that oestrogen might maintain PR expression in the stromal cells of uterine tumours. As a result, progesterone action at the molecular level would be enhanced, and uterine tumour cell proliferation would be suppressed.

The third and most important finding of the Janzen study was the identification of a link between the occurrence of progesterone resistance and epigenetic regulation of stromal PR expression. They found that only mice in which PTEN had been specifically deleted in epithelial cells showed a shift from progesterone sensitivity to progesterone resistance when stromal PR expression was decreased. Mice lacking both epithelial PTEN and Kirsten rat sarcoma (KRAS) viral oncogene homolog, a genetic mutation detected in women with EC, exhibited uterine tumour growth but failed to respond to progesterone treatment due to the absence of stromal PR. The mechanism behind the loss of stromal PR expression – especially stromal PRA – is due to enhanced DNA methylation of the stromal PR gene. These results suggest that under normal circumstances, the activities of PTEN and KRAS coordinate the progesterone response of endometrial cells.

Janzen *et al* have provided new insights into the mechanism by which the stromal component of the endometrium is responsible for progesterone sensitivity and resistance. Their work has demonstrated that PRA is a critical factor mediating the endometrial cellular response to progesterone treatment in EC tissues (Figure 1B). It is well known that the two PR isoforms form dimers *in vivo* upon their activation [46], and PRA and PRB are approximately evenly expressed in non-malignant areas of EC [23, 26]. However, the PR-mediated responses depend on the coordinated, opposing, and compensatory functions of the two PR isoforms [13, 47], and it has been shown that the activation of individual PR isoforms results in differential regulation of progesterone target genes [48]. In this context, future studies should be directed toward deciphering the role that PR isoform-specific signalling plays in the tumour–stroma interaction in response to progesterone treatment.

## Conclusion and future directions

Progesterone and PRs play a significant role in normal and neoplastic endometria [8–11], and specific PR isoforms in different endometrial cell populations might provide a molecular basis for the apparent relationship between progesterone sensitivity and resistance in women with early EC. In contrast to genetic mutations, DNA methylation is reversible. Therefore, increasing PRA or PRB expression by the epigenetic modulation might represent a promising therapeutic target for the treatment of EC in women with progesterone resistance.

Approximately 30% of women with polycystic ovary syndrome fail to respond to progesterone treatment and undergo progression to atypical hyperplasia and further transformation to EC [49]. The molecular mechanisms underlying endometrial progesterone resistance or sensitivity in these patients are not well understood, but the observations of Janzen *et al* help to explain why EC tissues respond the way they do to progesterone treatment *in vivo*. Because there are several morphological and physiological differences between rodent and human uteri, the challenge for future studies in this field is to determine whether the two PR isoform signalling intermediates play any role in progesterone resistance in the human endometrium under disease conditions. This is particularly important because for PRB signalling defects are not apparent [14]. We agree with the conclusion of Janzen *et al* that ‘defining mechanisms and site of origin for innate or acquired resistance to hormonal therapy in human EC trials will have immense translational application’ [44].

## Conflicts of interest

The author has no conflicts of interest to declare.
